# Elucidating different pattern of immunoregulation in BALB/c and C57BL/6 mice and their F1 progeny

**DOI:** 10.1038/s41598-020-79477-7

**Published:** 2021-01-15

**Authors:** Wiebke Hartmann, Birte Blankenhaus, Marie-Luise Brunn, Jana Meiners, Minka Breloer

**Affiliations:** 1 Helminth Immunology Group, Bernhard Nocht Institute for Tropical Medicine, Hamburg, Germany; 2 Department of Biology, University of Hamburg, Hamburg, Germany; 3 Instituto de Medicina Molecular João Lobo Antunes, Lisbon, Portugal

**Keywords:** Immunology, Immune evasion, Infection, Infectious diseases, Mucosal immunology

## Abstract

Helminths are large multicellular parasites that infect one quarter of the human population. To prolong their survival, helminths suppress the immune responses of their hosts. *Strongyloides ratti* delays its expulsion from the gut by induction of regulatory circuits in a mouse strain-specific manner: depletion of Foxp3^+^ regulatory T cells (Treg) improves the anti-*S. ratti* immunity in BALB/c but not in C57BL/6 mice. In the current study we compare the hierarchy of immunoregulatory pathways in BALB/c, C57BL/6 mice and their F1 progeny (BALB/c × C57BL/6). Using multicolor flow cytometry, we show that *S*. *ratti* induces a distinct pattern of inhibitory checkpoint receptors by Foxp3^+^ Treg and Foxp3^−^ T cells. Intensity of expression was highest in C57BL/6 and lowest in BALB/c mice, while the F1 cross had an intermediate phenotype or resembled BALB/c mice. Treg subsets expanded during infection in all three mouse strains. Similar to BALB/c mice, depletion of Treg reduced intestinal parasite burden and increased mucosal mast cell activation in *S. ratti*-infected F1 mice. Our data indicate that Treg dominate the regulation of immune responses in BALB/c and F1 mice, while multiple regulatory layers exist in C57BL/6 mice that may compensate for the absence of Treg.

## Introduction

Helminths are large multicellular parasites that cause chronic infections with an impaired protective immune response. *S. ratti* is a well-established murine model for intestinal nematode infections with tissue migrating stages. Infective third stage larvae (iL3) either actively penetrate the skin of their rodent host or are injected subcutaneously. Within 2 days the larvae migrate via incompletely defined pathways to the head and are swallowed. By day 3 post infection (p.i.) iL3 reach the intestine and develop via a fourth larval stage into female adults. Parasites live embedded in the mucosa of the intestine and reproduce via parthenogenesis by day 5 p.i. Eggs and already hatched first stage larvae are released in the environment with the feces. BALB/c and C57BL/6 mice both develop patent infections that are cleared in the context of a type 2 immune response after 2–4 weeks. While the kinetics of final clearance is similar, C57BL/6 mice usually display higher intestinal parasite burdens at the peak of infection compared to BALB/c mice^1–3^.

Helminths exploit their hosts’ immune regulatory pathways and induce the expansion of several regulatory cells and receptors in order to prolong their survival^4^. Regulatory T cells have been described to be critical in preventing severe immune pathology^5,6^ and in dampening the anti-helminth immune responses in mice and humans^3,7–12^. Regulatory T cells can be classified in CD4^+^ Foxp3^+^ Treg and IL-10 producing CD4^+^ Foxp3^−^ type 1 regulatory (Tr1) T cells. We have previously shown that *S. ratti* infection induces expansion of Foxp3^+^ Treg in BALB/c and C57BL/6 mice^3^. Using the depletion of regulatory T cells (DEREG) mouse model on these two genetic backgrounds, Treg depletion improves expulsion of *S. ratti* selectively in BALB/c mice, but not in C57BL/6 mice. Mechanistically, improved resistance in Treg-depleted BALB/c mice is mediated by increased IL-9-driven mast cell activation. Mast cell deficiency or IL-9 blockade, but not application of an anti-Gr-1 antibody in Treg-depleted BALB/c DEREG mice abrogates this phenotype^3^. Our data indicate that Treg control the activation of mast cells in BALB/c mice via regulation of IL-9, while Gr-1^+^ granulocytes such as neutrophils and eosinophils^13^ are dispensable. Despite increased type 2 immune responses in Treg-depleted C57BL/6 mice, the elevation of *S*. *ratti*-specific IL-9 production is not sufficient to trigger increased mast cell activation and accelerate parasite clearance, suggesting the presence of additional regulatory elements. Indeed, we observed that upregulation of the inhibitory receptor BTLA by T cells negatively regulates the anti-helminth immune response in *S. ratti*-infected C57BL/6 mice^14^. C57BL/6 mice either deficient for B and T lymphocyte attenuator (BTLA) or its ligand herpes virus entry mediator (HVEM) have reduced numbers of parasitic adults in the small intestine, reduced larval output throughout the infection as well as increased IL-9-driven mast cell activation.

The aim of the current study was to compare the hierarchy of immune regulatory pathways during infection with *S. ratti* in BALB/c, C57BL/6 mice and the F1 cross of both mouse strains (BALB/c × C57BL/6). Immune checkpoint molecules are regulators of the immune system and are crucial for the function of Treg populations. We analyzed the expression of the immune checkpoints cytotoxic T lymphocyte antigen-4 (CTLA-4), BTLA, programmed death-1 (PD-1), V-domain Ig-containing suppressor of T cell activation (VISTA), CD39, lymphocyte activation gene-3 (LAG-3), immunoglobulin-like transcript 3 (ILT3) and T cell immunoglobulin-3 (TIM-3) under steady state conditions and at the peak of *S. ratti* infection. CTLA-4 (CD152) binds with high affinity to CD80/CD86 and thereby prevents CD28-dependent co-stimulatory signals, while BTLA (CD272) delivers negative signals into the cell after binding to its ligand HVEM (CD270). The expression of both immune checkpoints is upregulated by CD4^+^ T cells from *S. ratti*-infected mice as we have previously shown^9,14^. PD-1 (CD279) and VISTA, also known as PD-H1, are non-redundant inhibitory effector molecules regulating T cells responses^15^. PD-1 binds to PD-L1 (CD274) or PD-L2 (CD273), while VISTA may either function as a ligand or as a receptor^16^. LAG-3 (CD223) binds with high affinity to MHC-II. This interaction prevents the binding of CD4 to MHC-II and directly blocks T cell activation^17^. In addition, LAG-3 in combination with CD49b identifies Tr1 cells^18^. CD39 is an ectonucleotidase that contributes to the suppressive capacity of Treg^19^. ILT3 (CD85k) is a transmembrane glycoprotein that contains intracellular immunoreceptor tyrosine-based inhibitory motifs. ILT3^+^ Treg have been described to promote the maturation of Th2 cells-inducing dendritic cells and thus are poor regulators of type II immunity^20,21^. Recently CD166 was identified as a ligand for ILT3^22^. Four Ligands (galectin-9, carcinoembryonic antigen cell adhesion molecule 1, Phosphatidylserine and high-mobility group protein B1) have been reported to bind to the receptor TIM-3 (CD366). TIM-3 lacks inhibitory motifs in its cytoplasmic region and has depending on the context co-stimulatory or co-inhibitory functions^23^.

Our analysis revealed distinct expression patterns in BALB/c, F1 and C57BL/6 mice. Thereby, the majority of the immune checkpoints including BTLA were highest in CD4^+^ Foxp3^+^ Treg and CD4^+^ Foxp3^−^ T cells from infected C57BL/6 mice and lowest in infected BALB/c mice. F1 mice either resembled the phenotype of BALB/c mice or showed intermediate expression levels compared to BALB/c and C57BL/6 mice. Similar to our previous data^3^ depletion of Treg resulted in an accelerated mast cell activation and expulsion of *S. ratti* in BALB/c, but not in C57BL/6 mice while the F1 mice phenocopied BALB/c mice. In summary, our data indicate that Foxp3^+^ Treg are central in the regulation of immune responses in BALB/c and the BALB/c x C57BL/6 F1 mice. By contrast, in C57BL/6 mice multiple layers of regulation exist that may compensate for the absence of Treg.

## Results

### Lower parasite burden in F1 and BALB/c mice than in C57BL/6 mice

**Figure 1:**
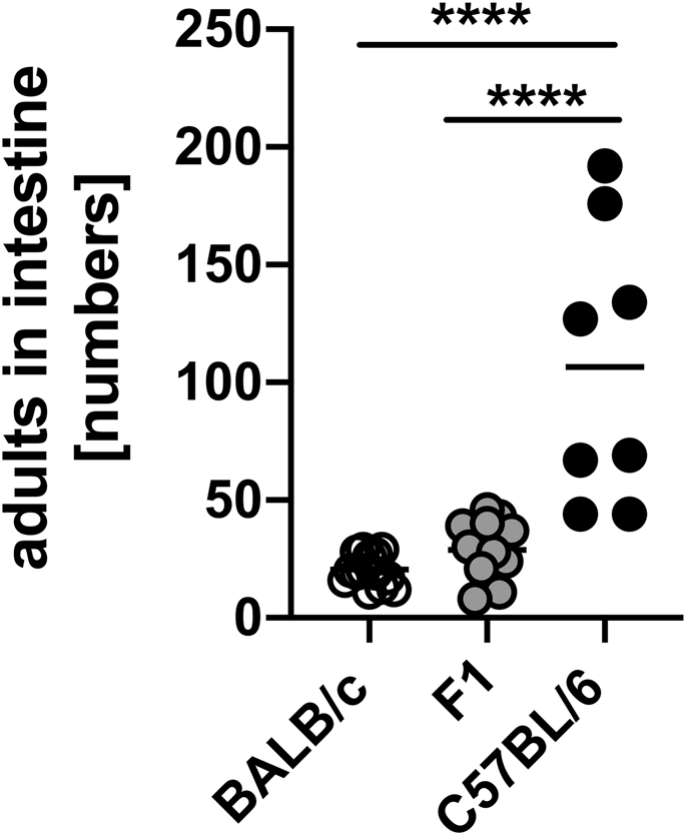
F1 mice have lower numbers of adults in the intestine than C57BL/6 mice*.* BALB/c, F1 and C57BL/6 mice were infected with 2000 iL3 s.c. in the footpad. Parasitic adults in the small intestine were counted at day 6 p.i. Shown are combined results from 2–3 experiments (BALB/c: n = 14 mice, F1: n = 13 mice and C57BL/6: n = 8 mice). Each symbol represents the worm burden from a single mouse. Horizontal lines represent the mean. Data were analyzed by 1-way ANOVA with Tukey's multiple comparisons test. Asterisks indicate significant differences of the mean (****p ≤ 0.0001).

The aim of the current study was to compare the regulation of the immune response against *Strongyloides ratti* infection in C57BL/6, and BALB/c mice and the F1 cross of both mouse strains (BALB/c × C57BL/6). Both parental mouse strains develop a patent *S. ratti* infection that is cleared with similar kinetics. However, C57BL/6 mice display higher intestinal worm burden at the peak of infection compared to BALB/c mice^1–3^. To quantify the susceptibility of their F1 cross we infected all three genotypes s.c. in the hind footpad with *S. ratti* and counted the number of parasitic adults at day 6 p.i. The number of adults in the intestine was significantly higher in C57BL/6 mice compared to BALB/c mice as observed before^1,3^. Parasite burden in F1 mice was similar to BALB/c mice (Fig. 1).

### Treg and Tr1 cell numbers increase during infection with *S. ratti*

**Figure 2:**
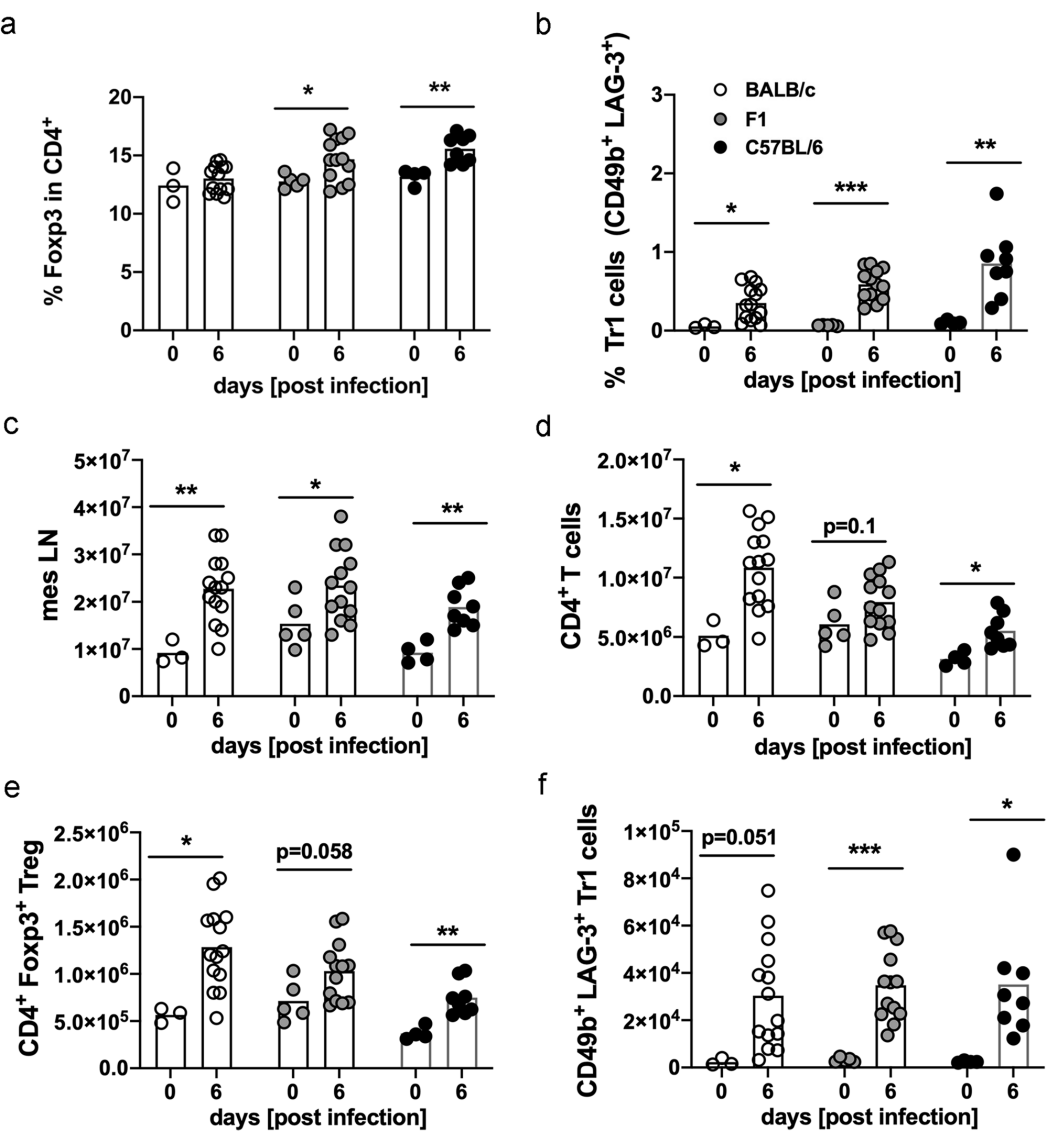
Treg and Tr1 cells increase during infection with *S. ratti. *BALB/c, F1 and C57BL/6 mice were left naïve (day 0) or infected with 2000 iL3. Six days later mes LN were isolated and stained with a panel for regulatory receptors. Frequencies of (**a**) Foxp3^+^ cells in CD4^+^ T cells and (**b**) Tr1 cells in Foxp3^−^ CD4^+^ T cells are shown. Numbers of mes LN (**c**), CD4^+^ T cells (**d**), Foxp3^+^ Treg (**e**), and CD49b^+^ LAG-3^+^ Tr1 cells (**f**). Each symbol represents values from a single mouse. Shown are combined results from 2–3 experiments (BALB/c day 0 p.i.: n = 3, day 6 p.i.: n = 14; F1 day 0 p.i.: n = 5, day 6 p.i.: n = 13; C57BL/6 day 0 p.i.: n = 4 mice, day 6 p.i.: n = 8). Data were analyzed by an unpaired t-test. Asterisks indicate significant differences of the mean (*p ≤ 0.05, **p ≤ 0.005, ***p ≤ 0.001).

We have previously shown that infection with *S. ratti* elevated the numbers of Treg^3^. We now compared the frequency of regulatory T cell subsets (Foxp3^+^ Treg and CD49b^+^ LAG-3^+^ Foxp3^−^ Tr1 cells) in the mesenteric lymph nodes (mes LN) from BALB/c, F1 and C57BL/6 mice (see gating strategy Supplementary Fig. 1). In line with our previous data infection of BALB/c mice with *S. ratti* did not alter the frequency of conventional Treg, defined as Foxp3^+^ T cells within the CD4^+^ T cells, while the frequency of Foxp3^+^ cells increased in infected F1 and C57BL/6 mice compared to non-infected mice (Fig. 2a). The frequency of Tr1 cells expanded in all three infected murine genotypes compared to the non-infected mice (Fig. 2b). Infection with *S. ratti* resulted in an increase of total mes LN cells in all three murine genotypes to a similar extent (Fig. 2c). Thereby numbers of CD4^+^ T cells in the mesLN increased during infection in BALB/c (average number: 1.0 × 10^7^ cells) and C57BL/6 mice (5.5 × 10^6^ cells) and by trend (p = 0.1) in F1 mice (average number: 7.9 × 10^6^ cells). *S. ratti*-infected C57BL/6 mice displayed the lowest numbers of CD4^+^ T cells (Fig. 2d) compared to infected BALB/c and F1 mice. In line with this infected BALB/c and F1 mice displayed slightly higher numbers of Treg (BALB/c: 1.2 × 10^6^ Treg, F1: 1.0 × 10^6^ Treg) in the mes LN compared to infected C57BL/6 mice (7.4 × 10^5^ Treg) (Fig. 2e). The number of Tr1 cells (Fig. 2f) increased in infected BALB/c, F1 and C57BL/6 mice compared to non-infected mice, although due to high variance it did not reach statistical significance in BALB/c mice (p = 0.051).

### Distinct expression patterns by Treg and Foxp3^−^ T cells from BALB/c, F1 and C57BL/6 mice

**Figure 3:**
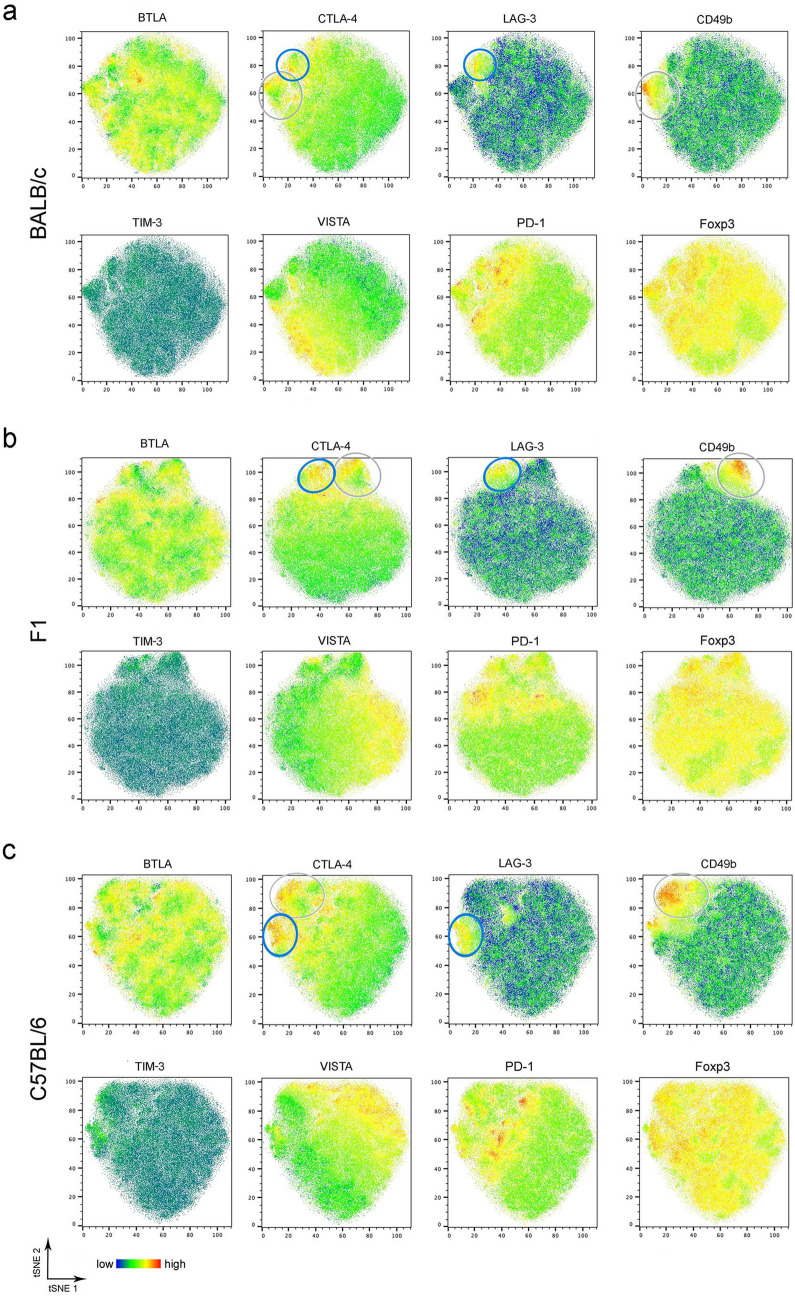
Similar expression pattern of regulatory markers by Treg from BALB/c, F1 and C57BL/6 mice. BALB/c, F1 and C57BL/6 mice were infected with 2000 iL3. Six days later mes LN were isolated and stained with a panel for regulatory receptors. t-SNE calculation was performed with 40,000 Foxp3^+^ CD4^+^ Treg using FlowJo Plugins. Representative t-SNE heatmaps derived from Treg from BALB/c (**a**), F1 (**b**) and C57BL/6 (**c**) showing the expression of BTLA, CTLA-4, LAG-3, CD49b, TIM-3, VISTA, PD-1 and Foxp3.

**Figure 4:**
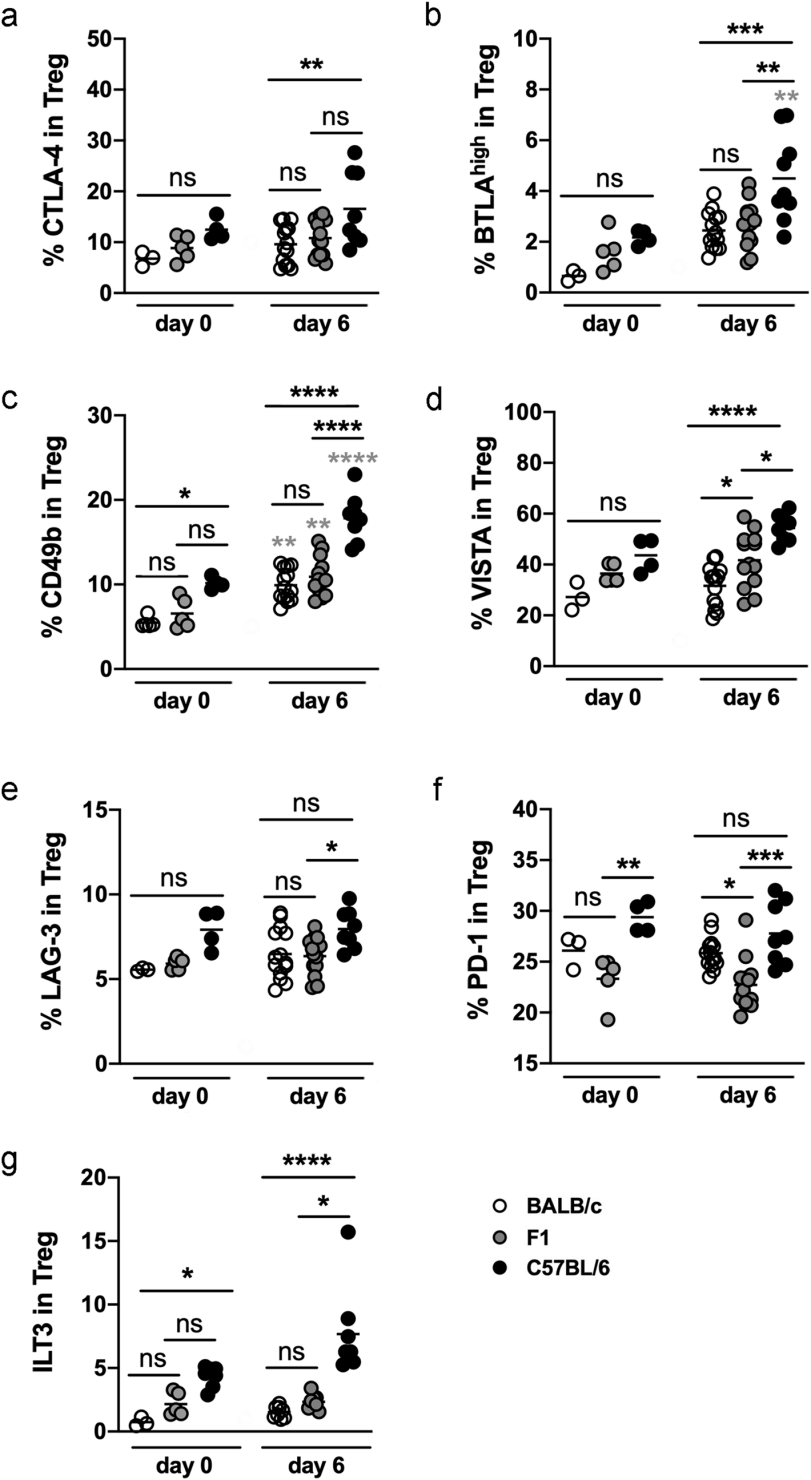
Increased expression of distinct checkpoint molecules by Treg from C57BL/6 mice. BALB/c, F1 and C57BL/6 mice were left naïve (day 0) or infected with 2000 iL3. Six days later mes LN were isolated and stained with a panel for regulatory receptors (**a–f**) or ILT3 (**g**). Statistical analysis showing the expression of CTLA-4 (**a**), BTLA^high^ (**b**), CD49b (**c**), VISTA (**d**), LAG-3 (**e**), PD-1 (**f**), and ILT3 (**g**) by Treg. The gating strategy is shown in the Supplementary Figs. S1 and S2. Each Symbol represents one mouse. Shown are combined results from 2 to 3 experiments (a-f: BALB/c day 0 p.i.: n = 3, day 6 p.i.: n = 14; F1 day 0 p.i.: n = 5, day 6 p.i.: n = 13; C57BL/6 day 0 p.i.: n = 4 mice, day 6 p.i.: n = 8; g: BALB/c day 0 p.i.: n = 3 mice; F1 day 0 p.i.: n = 5 mice; C57BL/6 day 0 p.i.: n = 5 mice; n = 8 mice for all infected mouse strains). Data (**a–f**) were analyzed by 1-way ANOVA with Tukey's multiple comparisons test. Differences in the ILT3 expression (**g**) were analyzed by Kruskal–Wallis test. Black asterisks are shown for naïve and infected mice and indicate significant differences of the mean (*p ≤ 0.05, **p ≤ 0.005, ***p ≤ 0.001, ****p ≤ 0.0001, ns = non significant). If in a graph only a large bar with ns is depicted, no statistical differences between the three genotypes exist at this time point. Grey asterisks indicate significant differences between day 0 and day 6 p.i. from one murine genotype.

Using multicolor flow cytometry, we next analyzed the expression of the inhibitory immune checkpoint molecules CTLA-4, BTLA, PD-1, VISTA, LAG-3, and TIM-3 by Foxp3^+^ Treg in the mes LN. First, we performed an unbiased t-distributed stochastic neighbor embedding (t-SNE) analysis to visualize cell clusters within the Foxp3^+^ Treg (Fig. 3). T-SNE is a dimensionality reduction plugin from FlowJo, that was applied to pre-gated CD4^+^ Foxp3^+^ T cells. Dead cells and doublets were excluded (see gating strategy Supplementary Fig. 2). The generated t-SNE plots from Treg did not reveal distinct populations in BALB/c, C57BL/6 and F1 mice (Fig. 2). We observed a coexpression of the marker CTLA-4 with CD49b (Fig. 3, grey gate) or LAG-3 (Fig. 3, blue gate) in all mouse strains, while all other markers showed a patchy distribution. Expression of VISTA or PD-1 only partially overlapped with other markers. The majority of PD-1^+^ or VISTA^+^ Treg did not coexpress CTLA-4, BTLA, LAG-3 or CD49b. Strikingly, Treg from C57BL/6 mice showed an increased expression of CTLA-4, CD49b and VISTA compared to Treg from F1 mice and even more pronounced compared to Treg from BALB/c mice, which was confirmed by statistical analysis (Fig. 4). The receptor TIM-3 was barely detectable (Supplementary Fig. 2b) and expression levels were similar by Treg from BALB/c, F1 and C57BL/6 mice (Fig. 3). Statistical analysis of Foxp3^+^ Treg showed the highest expression of CTLA-4 (Fig. 4a), BTLA^high^ (Fig. 4b), CD49b (Fig. 4c), and VISTA (Fig. 4d) in C57BL/6 mice compared to BALB/c mice. Mes LN from F1 mice exhibited an intermediate phenotype or were similar to BALB/c mice at day 0 and day 6 p.i. Murine genotype or infection did not alter the expression level of LAG-3 by Treg (Fig. 4e). Surprisingly, the expression of PD-1 did not increase during infection with *S. ratti* (Fig. 4f). Constitutive PD-1 expression was lowest in mes LN from F1 mice and significantly higher in Treg derived from *S. ratti*-infected BALB/c and C57BL/6 mice. Next, we analyzed the expression of ILT3 (gating strategy Supplementary Fig. 3). ILT3 expression by Foxp3^+^ Treg from C57BL/6 mice was already higher at steady state (day 0) (Fig. 4g). During infection ILT3 increased only slightly in C57BL/6 mice, but was significantly higher compared to infected BALB/c and F1 mice. By contrast, infection with *S. ratti* did not alter the ILT3 expression by Treg from BALB/c and F1 mice.

**Figure 5:**
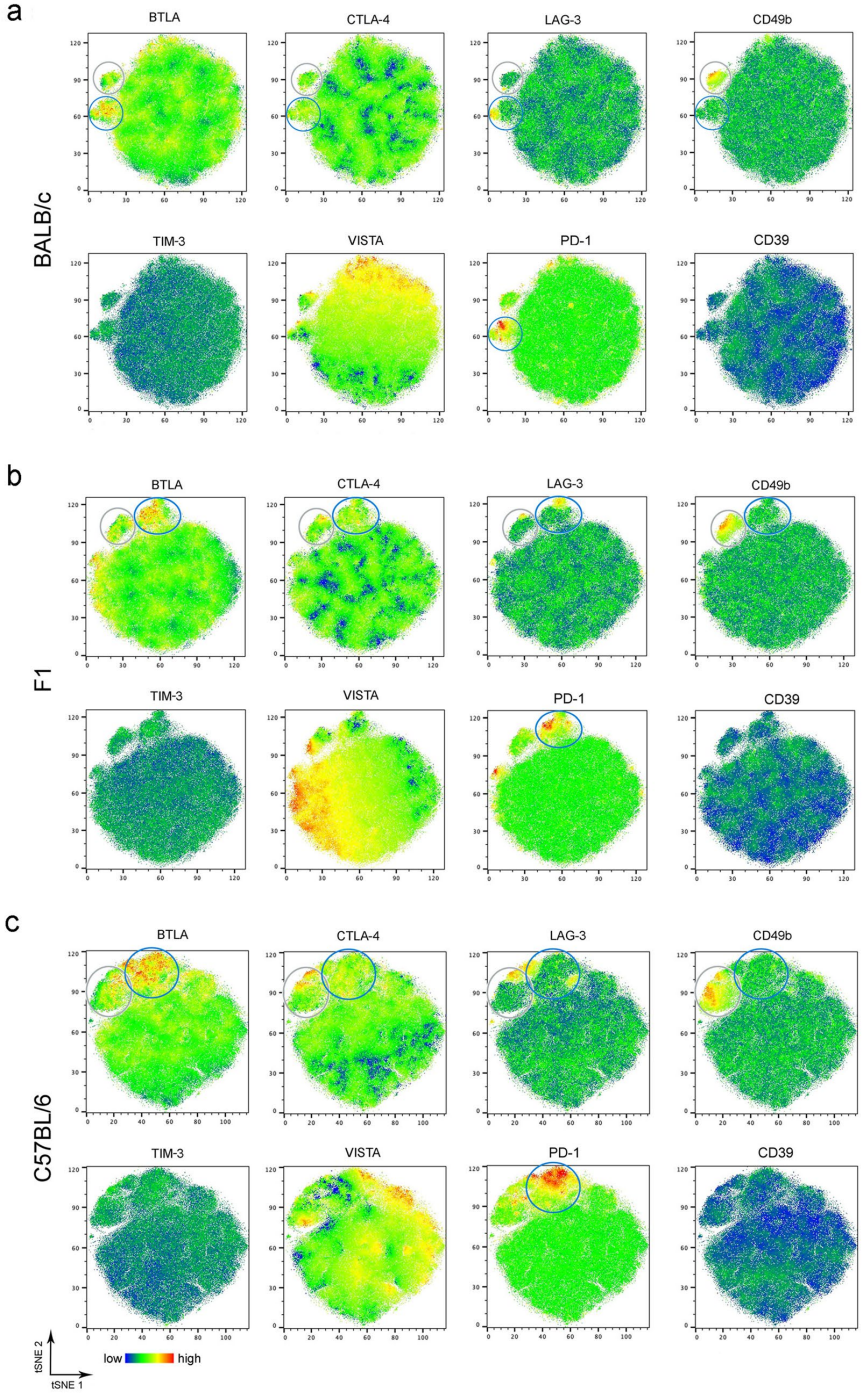
Expression of regulatory receptors is more pronounced by Foxp3^−^ CD4^+^ T cells from C57BL/6 mice than BALB/c and F1 mice. BALB/c, F1 and C57BL/6 mice were infected with 2000 iL3. Six days later mes LN were isolated and stained with a panel for regulatory receptors. t-SNE calculation was performed with 80,000 Foxp3^−^CD4^+^ T cells using FlowJo Plugins. Representative t-SNE heatmaps derived from BALB/c (**a**), F1 (**b**) and C57BL/6 (**c**) showing the expression of BTLA, CTLA-4, LAG-3, CD49b, TIM-3, VISTA, PD-1 and CD39.

**Figure 6:**
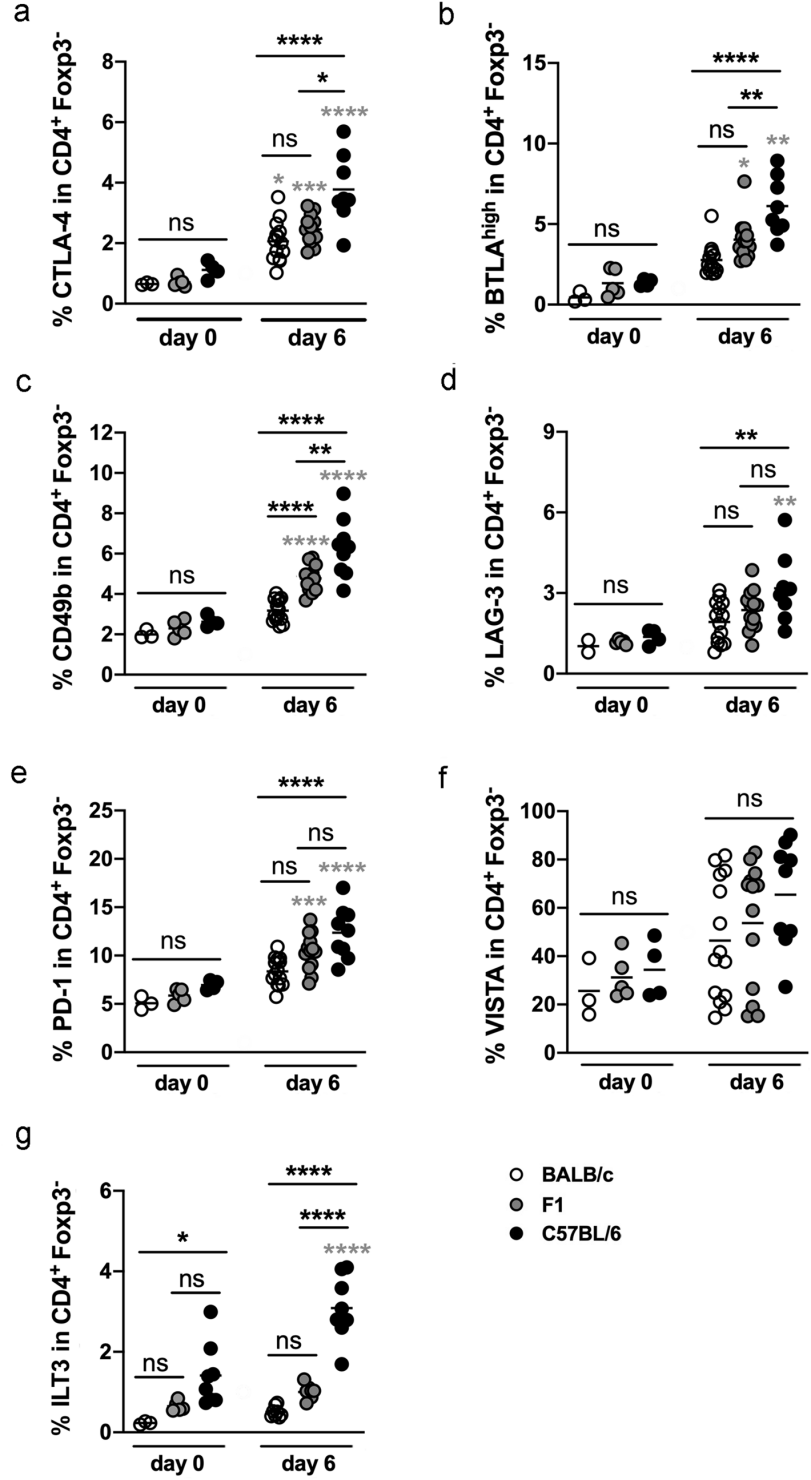
Highest expression of regulatory receptors by Foxp3^−^ T cells from C57BL/6 mice. BALB/c, F1 and C57BL/6 mice were left naïve (day 0) or infected with 2000 iL3. Six days later mes LN were isolated and stained with a panel for regulatory receptors (**a**–**f**) or for ILT3 (**g**). Statistical analysis showing the expression of CTLA-4 (**a**), BTLA^high^ (**b**), CD49b (**c**), LAG-3 (**d**), PD-1 (**e**), VISTA (**f**), and ILT3 (**g**) by CD4^+^ Foxp3^−^ CD4^+^ T cells. Each Symbol represents one mouse. Shown are combined results from 2 to 3 experiments (**a**–**f**: BALB/c day 0 p.i.: n = 3, day 6 p.i.: n = 14; F1 day 0 p.i.: n = 5, day 6 p.i.: n = 13; C57BL/6 day 0 p.i.: n = 4 mice, day 6 p.i.: n = 8; **g**: BALB/c day 0 p.i.: n = 3 mice; F1 day 0 p.i.: n = 5 mice; C57BL/6 day 0 p.i.: n = 8 mice; n = 8 mice for all infected mouse strains). Data (**a**,**c**,**d**,**e**,**g**) were analyzed by 1-way ANOVA with Tukey's multiple comparisons test; panel b and f were analyzed by Kruskal–Wallis test. Black Asterisks indicate differences of the mean between naïve or infected mice and indicate significant differences (**p ≤ 0.005, ***p ≤ 0.001, ****p ≤ 0.0001, ns = non significant). If in a graph only a large bar with ns is depicted, no statistical differences between the three genotypes exist at this time point. Grey asterisks indicate significant differences between day 0 and day 6 p.i. from one murine genotype.

T-SNE analysis from CD4^+^ Foxp3^−^ T cells revealed more pronounced differences in the expression levels of all checkpoint receptors between the three murine genotypes (Fig. 5a–c). We observed coexpression of the markers CD49b and LAG-3 (Fig. 5, grey gate) indicating the presence of Tr1 cells. CD49b^+^ LAG3^+^ Tr1 cells were partially positive for CTLA-4 and BTLA (Fig. 5, grey gate). The majority of LAG-3^+^ Foxp3^−^ T cells did not coexpress CD49b (Fig. 5, blue gate). Cells with the highest BTLA expression coexpressed PD-1 and fractions were positive for LAG-3 and CTLA-4 (Fig. 5, blue gate). Foxp3^−^ T cells from all mouse strains were highly positive for VISTA. The expression of the markers TIM-3 and CD39 was low in BALB/c, F1 and C57BL/6 mice (Fig. 5 and Supplementary Fig. 2b). The statistical analysis confirmed that the expression of CTLA-4 (Fig. 6a), BTLA^high^ (Fig. 6b), CD49b (Fig. 6c), LAG-3 (Fig. 6d), and PD-1 (Fig. 6e) was highest in C57BL/6 mice and lowest in BALB/c mice. Foxp3^−^ T cells from F1 mice had intermediate expression levels of these markers. VISTA expression was similar in all mouse strains and was not upregulated compared to non-infected control mice (Fig. 6f). As shown for Foxp3^+^ Treg (Fig. 4g) ILT3 expression by Foxp3^−^ T cells from C57BL/6 mice was already higher at steady state (Fig. 6g) compared to BALB/c mice and by trend to F1 mice. The expression of ILT3 increased during infection in C57BL/6 mice and was significantly higher compared to infected BALB/c and F1 mice while the expression remained unchanged by Foxp3^−^ T cells from BALB/c and F1 mice.

In summary, analysis of immune checkpoints revealed distinct expression patterns by Foxp3^+^ Treg and Foxp3^−^ T cells during infection and already by trend under steady state conditions. Strikingly, T cells from C57BL/6 mice expressed significantly higher levels of the majority of immune checkpoints compared to BALB/c mice. T cells from F1 mice either phenocopied the expression levels from BALB/c mice or showed an intermediate expression. Parasite burden from F1 mice resembled BALB/c mice as well. In line with the more pronounced layers of regulation, C57BL/6 mice had increased numbers of adults in the intestine compared to BALB/c and F1 mice.

### Treg are central in the regulation of the anti-helminth immune response in F1 mice

**Figure 7:**
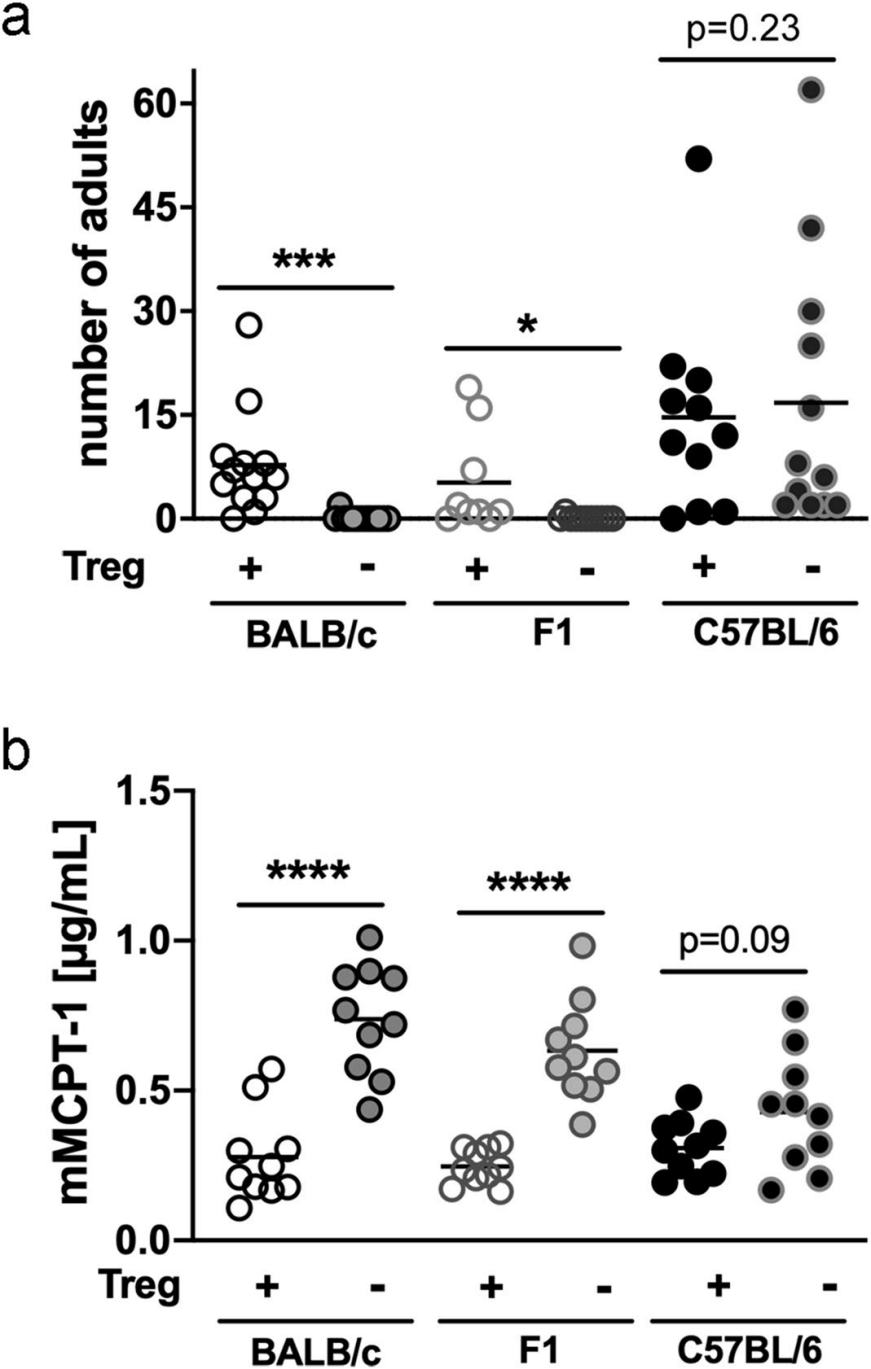
Depletion of Treg in F1 mice results in improved expulsion of *S. ratti* from the intestine. BALB/c DEREG, F1 DEREG and C57BL/6 DEREG mice and their littermates were infected with 2000 iL3 s.c. in the footpad. DEREG mice (− Treg) and their non-transgenic littermates (+ Treg) were treated with 0.5 µg DT one day prior to *S. ratti* infection and on the 2 following days. (**a**) Parasitic adults in the small intestine were counted at day 6. Shown are combined results from 3 experiments (BALB/c + /− Treg: n = 13; F1 + Treg: n = 9, F1 − Treg: n = 11; C57BL/6 + Treg: n = 11, C57BL/6 − Treg: n = 12). (**b**) Concentration of mMCPT-1 in the serum of day 5 infected mice. Each symbol represents the worm burden and the amount of mMCPT-1 from a single mouse. Horizontal lines represent the mean. Shown are combined results from 3 different experiments with n = 10 mice per group. Data were analyzed by unpaired t-test comparing mice with and without Treg from one genotype. Asterisks indicate significant differences of the mean (*p ≤ 0.05, ***p ≤ 0.001, ****p ≤ 0.0001).

Since we have previously shown that Treg significantly suppress the anti-*S. ratti* immune response in BALB/c mice^3^, we now aimed to compare the impact of Foxp3^+^ Treg in BALB/c and C57BL/6 mice and their F1 generation. To this end, we made use of the DEREG mice, which allow the depletion of Treg by application of diphtheria toxin (DT)^24^. To generate F1 DEREG mice, C57BL/6 DEREG mice were crossed with BALB/c mice and vice versa. We ended up with two different genotypes, F1 mice with heterozygous expression of the DT receptor and F1 littermates that did not express the transgene. All groups were treated with DT and subsequently infected with *S*. *ratti*. Transient Treg depletion resulted in improved eradication of *S. ratti* in BALB/c DEREG mice while C57BL/6 DEREG mice did not benefit from Treg depletion (Fig. 7a) as we have shown before^3^. The reduced number of worms in Treg-depleted BALB/c mice was accompanied by an increased mast cell degranulation (Fig. 7b). The amount of the mast cell-specific mouse mast cell protease-1 (mMCPT-1) in the serum of infected C57BL/6 mice was comparable in Treg-depleted and non-depleted DEREG mice. The Treg-depleted DEREG F1 generation of BALB/c and C57BL/6 mice had reduced parasite burden (Fig. 7A) and increased mast cell degranulation (Fig. 7b) and thus phenocopied the Treg-depleted BALB/c mice.

Taken together our data show a similar phenotype of F1 and BALB/c mice. In both mouse strains the anti-*S. ratti* response is controlled by Treg as non-redundant suppressor cells, while in C57BL/6 mice depletion of Treg did not increase mast cell-mediated expulsion of *S. ratti.* In line with the missing impact of Treg depletion, CD4^+^ T cells from C57BL/6 mice displayed a distinct and more pronounced expression of immune checkpoint molecules compared to BALB/c and F1 mice.

## Discussion

Inbred mouse strains such as C57BL/6 and BALB/c mice are valuable models to study immune responses during infectious diseases^25^. These mouse strains are well defined and various cell- and receptor-deficient mice exist on both genetic backgrounds. In the current study we compared the regulation of the immune response against the intestinal parasite *S. ratti* in C57BL/6, BALB/c mice and the F1 generation “hybrid” (BALB/c × C57BL/6) of both mouse strains. In line with previous findings^1–3^ we recorded increased parasite burden in C57BL/6 mice compared to BALB/c mice at the peak of infection. Interestingly, the F1 cross resembled BALB/c mice with regard to parasite burden.

Performing a comprehensive flow cytometric analysis, we recorded the expression of a variety of immune checkpoint molecules in these three distinct genetic backgrounds. Mice were analyzed under steady state conditions as well as during acute infection with the gastrointestinal nematode *S. ratti*. We identified pronounced differences in the expression pattern of immune checkpoints by Foxp3^+^ and Foxp3^−^ CD4^+^ T cells derived from BALB/c and C57BL/6 mice already at steady state. Despite a similar kinetic of *S. ratti* expulsion^1–3^, our data clearly indicate differences in the regulation of immune responses in these two commonly used laboratory mouse strains. We show that the regulatory pattern of the F1 cross from BALB/c and C57BL/6 mice resembled BALB/c mice rather than C57BL/6 mice since (i) the expression of regulatory receptors by Foxp3^+^ Treg and Foxp3^−^ T cells in F1 mice mimics more the phenotype of BALB/c mice than C57BL/6 mice and (ii) depletion of Treg causes an improved mast cell activation and subsequent eradication of *S. ratti* in F1 and BALB/c mice, but not in C57BL/6 mice. We hypothesize that the pronounced expression of checkpoint receptors by Foxp3^−^ T cells or other regulatory cells subsets such as Tr1 cells compensates for the depletion of Foxp3^+^ Treg in C57BL/6 mice. The lack of phenotypic outcome of the Treg depletion with regard to parasite burden in C57BL/6 mice would emphasize the enhanced regulatory capacity of the Foxp3^−^ T cell populations to interfere with anti-*S. ratti* immune responses.

The impact of Tregs on anti-helminth immune responses was recently summarized^26^, however, there is no consistent analysis comparing the impact of the genetic murine background on the role of Treg during helminth infection. Tregs promote parasite survival during infection with *Schistosoma japonicum*^27,28^*, S. ratti*^3,9^*, Trichuris muris*^8^ and *Litomosoides sigmodontis*^11,29,30^ whereas they are redundant suppressor cells during infection with *Heligmosomoides polygyrus*^5,31^ and *Trichinella spiralis*^32^*.* Differences in the regulation might, however, not be parasite-specific but rely on the genetic background of the mice. Two recent studies compared the recruitment of Treg during infection with the gastrointestinal nematode *H. polygyrus*^31^ and the filarial nematode *L. sigmodontis*^11^ in BALB/c and C57BL/6 mice. Both studies did not compare the impact of early Treg depletion on the course of helminth infection. Supporting our data *H. polygyrus-*infected susceptible C57BL/6 mice had increased Foxp3^+^ Treg frequencies compared to more resistant BALB/c mice, while the total number of Helios^+^ Treg was higher in BALB/c mice. By contrast, a similar recruitment of Treg was shown for both genotypes during infection with *L. sigmodontis*. In this helminth model, BALB/c mice are susceptible to *L. sigmodontis* and develop patent, chronic infections, while C57BL/6 mice are semi-resistant and clear the infection before the onset of microfilaremia. Interestingly, CD4^+^ Foxp3^−^ T cells from *L. sigmodontis*-infected C57BL/6 mice had a more activated phenotype than T effector cells from BALB/c mice^11^. We observed an elevated frequency of CD49b^+^ LAG-3^+^ Tr1 cells and Foxp3^+^ Treg in CD4^+^ T cells and overall more activated phenotype of Foxp3^−^ CD4^+^ T cells in C57BL/6 mice compared to BALB/c and F1 mice. The number of regulatory T cells, however, was not higher in C57BL/6 mice due to lower total numbers of CD4^+^ T cells in C57BL/6 mice.

We now provide evidence that the F1 progeny after crossing BALB/c and C57BL/6 mice has a more BALB/c-like phenotype: BALB/c and F1 mice displayed similarly lower numbers of adults in the small intestine, and Treg depletion reduced intestinal parasite burdens and increased mast cell degranulation selectively in BALB/c and F1 mice, but not in C57BL/6 mice. Expulsion of *S. ratti* relies in both, BALB/c and C57BL/6 mice, on mast cells^2^ and on IL-9 that promotes mast cell activation early in infection^1^. However, the mechanisms controlling this crucial mast cell degranulation seem to be different in BALB/c, and C57BL/6 mice. In BALB/c mice Foxp3^+^ Treg suppress sufficient IL-9 production for subsequent mast cell degranulation during *S. ratti* infection in a non-redundant manner, whereas additional regulatory pathways seem to suppress mast cell activation in Treg-depleted C57BL/6 mice^3^.

The analysis of immune checkpoints that we performed in this study revealed striking differences in the expression pattern by T cells in the mesenteric lymph nodes, draining the site of infection in C57BL/6 mice compared to both BALB/c and F1 mice. We observed an increased expression of ILT3 selectively in C57BL/6 mice. ILT3 belongs to the immunoglobulin superfamily and contains immunoreceptor tyrosine-based inhibitory motifs suggesting an inhibitory function of ILT3^33^. Indeed, Tregs expressing ILT3 show lower T cell receptor signaling than ILT3^−^ Treg and favor the expansion of Th2 cell-inducing dendritic cells^20^. ILT3 is expressed by Foxp3^+^ Treg^20^ and in hyporesponsive Th2 cells^34^ during infection with *L. sigmodontis*. In this study hyporesponsive Th2 cells were distinguished from exhausted/anergic cells due to their specific gene expression pattern, which includes LAG-3^34^. We observe the highest expression of ILT3, LAG-3 and several other inhibitory receptors such as BTLA by Foxp3^−^ T effector cells from C57BL/6 mice while T cells in the mes LN from BALB/c and F1 mice showed a lower expression. BTLA is a non-redundant regulator of *S. ratti*-induced immune evasion in C57BL/6 mice. Mice lacking BTLA or its ligand HVEM have a reduced intestinal parasite burden and accelerated mast cell degranulation compared to wildtype mice^14^. We now report that Foxp3^+^ Treg and especially Foxp3^−^ CD4^+^ T cells from infected C57BL/6 mice display a significantly higher expression of BTLA than BALB/c and F1 mice which is in line with the non-redundant function of BTLA in suppressing anti-helminth immunity in C57BL/6 mice.

Infection with *S. ratti* did not induce a significant upregulation of TIM-3, CD39 or VISTA in BALB/c, C57BL/6 or F1 mice. Since the expression of the markers TIM-3 and CD39 has been described to occur under inflammatory conditions^35–37^ the missing expression in a helminth-induced Th2 environment is not unexpected. VISTA is a recently discovered immune checkpoint molecule that is constitutively expressed by T cells^38^. We show an expression of VISTA by Foxp3^+^ Treg and Foxp3^−^ Teff under steady state conditions. VISTA was not upregulated by Foxp3^−^ CD4^+^ T cells during infection in any of the three mouse strains indicating a minor role for VISTA in the regulation of anti-helminth immune responses. In summary, the missing expression of CD39, TIM-3 and VISTA shows that *S*. *ratti* did not induce broad unspecific upregulation of all immune checkpoints but rather led to a distinct regulatory pattern.

In general, immune checkpoints are expressed by activated T cells, Treg or exhausted T cells, i.e. cells that are persistently exposed to antigen during chronic infection and lose their functional capabilities^39^. In the current study, mice were acutely infected for 6 days with *S. ratti* at the time point of analysis. Thus, increased expression of checkpoint molecules by Foxp3^−^ CD4^+^ T cells in C57BL/6 mice compared to BALB/c or F1 mice is unlikely due to T cell exhaustion observed in chronic infections. Our flow cytometry data more likely identify activated T cells expressing inhibitory receptors suggesting redundant layers of regulation in C57BL/6 mice.

Helminths, employ multiple regulatory pathways of their host to modulate the immune response and to ensure their survival. In the current study we use a helminth that causes patent infections in BALB/c and C57BL/6 mice to point out that different genetically determined patterns of regulation exist in both mouse strains. These patterns were partially visible already at steady state and expanded during helminth infection. Thereby, the strain-specific differences that became apparent should be considered analyzing host–pathogen interactions. Using infections with *S. ratti* as a model for intestinal helminth infections, BALB/c traits are partially dominant and inherited to F1 mice in a controlled and optimized environment such as a “specific pathogen free” animal facility while in C57BL/6 mice multiple layers of regulation exist that may compensate for the absence of single regulatory circuits. The exact genetic mechanisms, and a putative contribution of X-linked dominant inheritance, causing the differences in the regulation of immune responses remain unknown and should be addressed in future studies.

Inbred mouse strains are homozygous and thus reflect only a minority of the genetic variation present in wild mice and in the human population. In the wild, susceptibility to infection is additionally influenced by demographic factors such as gender, age, and infection history^40^. It was previously shown that the immune status of C57BL/6 mice greatly differs from wild mice^41–43^ and is affected by body conditions and age and to a lesser extent by the genetic variations^44^. Consequently, data obtained using these two inbred mouse strains cannot be transferred directly to genetically more heterogeneous humans. A thorough analysis of different regulatory pattern in laboratory mouse strains may allow a better understanding of the variability observed in human studies in terms of helminth-induced immunomodulation.

## Material and methods

### Ethics and mice

Animal experiments were conducted in agreement with the German animal protection law. Experimental protocols were approved by Federal Health Authorities of the State of Hamburg with the permission numbers 54/10 and N103/2018. BALB/c, C57BL/6, F1 (BALB/c × C57BL/6) mice, BALB/c DEREG, C57BL/6 DEREG and F1 DEREG mice were bred at the animal facility of the BNITM and kept in individually ventilated cages under specific pathogen free conditions. For the generation of the F1 mice, female BALB/c mice were crossed with male C57BL/6 mice. To generate F1 DEREG mice, male C57BL/6 DEREG mice were crossed with female BALB/c mice and vice versa. DEREG mice express the DT receptor and the enhanced green fluorescence protein (eGFP) under the control of the Foxp3 promotor^24^. To deplete Foxp^+^ eGFP^+^ Treg and to control for side effects induced by DT, DEREG mice and their non-transgenic littermates were treated with 0.5 µg DT (Merck) in 200 µL PBS (pH 7.4) i.p. one day prior to *S. ratti* infection and on the 2 following days. Treg depletion was routinely controlled at day 2 p.i. by analysis of peripheral blood samples for eGFP and CD4 expression. For all experiments, female mice were used at 7–10 weeks of age and experimental groups were matched for age. For the flow cytometry data, one naïve C57BL/6 male mouse was used in addition to the female control mice.

### *S. ratti* infection and analysis of parasite burden

The *S. ratti* cycle was maintained in Wistar rats as described^45^ and infections were performed by s.c. injection of 2000 iL3 in the hind footpad of the mice. To count the number of female adults, the intestine was opened longitudinally and the feces was removed by flushing the small intestine with tap water. After 3 h incubation at 37 °C the released female adults were counted under the microscope.

### mMCPT-1 ELISA

For analysis of mMCPT-1, blood was collected from infected mice at day 5 p.i. and allowed to coagulate for 1 h at room temperature (RT). Serum was collected after centrifugation (10,000×*g*) for 10 min at RT. The mMCPT-1 concentration was quantified using MCPT-1 Ready-SET-Go kit (Thermofisher Scientific) according to the manufacturer’s recommendations.

### Flow cytometry staining of T cells

Single cells (3 × 10^6^) from the mesenteric lymph nodes were stained with 1 µL Zombie Yellow Fixable Viability Kit (Biolegend) in 1 mL PBS for 30 min at 4 °C. For surface staining, cells were first stained for 15 min at 37 °C with anti-mouse APC-labeled anti-mouse LAG-3 (clone: C9b7W) and PE/Cy7-labeled anti-mouse CD49b (clone: HMa2). After an additional incubation for 15 min at RT, cells were washed and stained for 30 min on ice with FITC-labeled antibodies against mouse PD-1 (clone: 29F.1A12), PE-labeled anti-mouse BTLA (clone: 6F7), PE/Dazzle 594-labeled anti-mouse VISTA, BV421-labeled anti-mouse TIM-3 (clone: RMT3-23), BV510-labeled anti-mouse CD4 (clone RM4-5), and PE-labeled ILT3 (CD85k, clone: H1.1). For subsequent intracellular staining with anti-mouse AF700-labeled anti-mouse Foxp3 (clone: FJK-16S) and PerCP Cy5.5-labeled anti-mouse CTLA-4 (clone: UC10-4B9), cells were fixed and permeabilized with Thermofisher Scientific Foxp3/Transcription factor staining buffer set according to the manufacturer’s protocol. Samples were analyzed on a LSRFortessa (Becton Dickinson) using FlowJo software (TreeStar). For the cluster analysis data were analyzed using t-SNE, a dimensionality reducing plugin for Flow Jo Version 10. The gating strategy is shown in Supplementary Fig. 2. The percentage of dead cells was always below 5%. Dead cells and doublets were excluded for the analysis. Cells were further distinguished by their expression of CD4 and Foxp3. Foxp3^−^ CD4^+^ T cells were downsampled to 80,000 and CD4^+^ Foxp3^+^ single cells were downsampled to 40,000 events per sample. Representative Heatmaps from the t-SNE analysis show the median expression intensity of Foxp3, PD-1, LAG-3, CD49b, BTLA, CTLA-4, VISTA, CD39, and TIM-3.

### Statistical analysis

Data were analyzed using Graph Pad Prism Version 8 and tested for normality distribution using Anderson–Darling test, D’Agostino and Pearson test and Shapiro–Wilk test. Comparing two groups to each other Student t-test (parametric) or Mann–Whitney-U test (non-parametric) was applied to the data. For comparison of more than two groups either 1-way ANOVA with Tukey’s multiple comparison test (parametric) or Kruskal–Wallis with Dunn’s multiple comparison test (non-parametric) were performed. Statistical tests are indicated in the Figure legends. Asterisks for all analysis *p ≤ 0.05, **p ≤ 0.01, ***p ≤ 0.001, ****p ≤ 0.0001.

## Supplementary Information

Supplementary Information.
