# Supplementary material for: Elucidating different pattern of immunoregulation in BALB/c and C57BL/6 mice and their F1 progeny
Source: Sci Rep. 2021 Jan 15;11:1536. doi: 10.1038/s41598-020-79477-7 (PMC7810711; doi:10.1038/s41598-020-79477-7)
Supplement: Supplementary file 1 — Supplementary Information. [file 41598_2020_79477_MOESM1_ESM.docx]

Supplementary Information

Elucidating different pattern of immunoregulation in BALB/c and C57BL/6 mice and their F1 progeny

**Wiebke Hartmann**^1*^**, Birte Blankenhaus**^1^**^,^** ^§^**, Marie-Luise Brunn**^1^**, Jana Meiners**^1^**, Minka Breloer**^1, 2^

^1^Helminth Immunology Group, Bernhard Nocht Institute for Tropical Medicine, Hamburg, Germany

^2^Department of Biology, University of Hamburg, Hamburg, Germany

^§^ present address: Instituto de Medicina Molecular João Lobo Antunes, Lisbon, Portugal

*** Correspondence:**Wiebke Hartmann
[hartmann@bnitm.de](mailto:hartmann@bnitm.de)

| 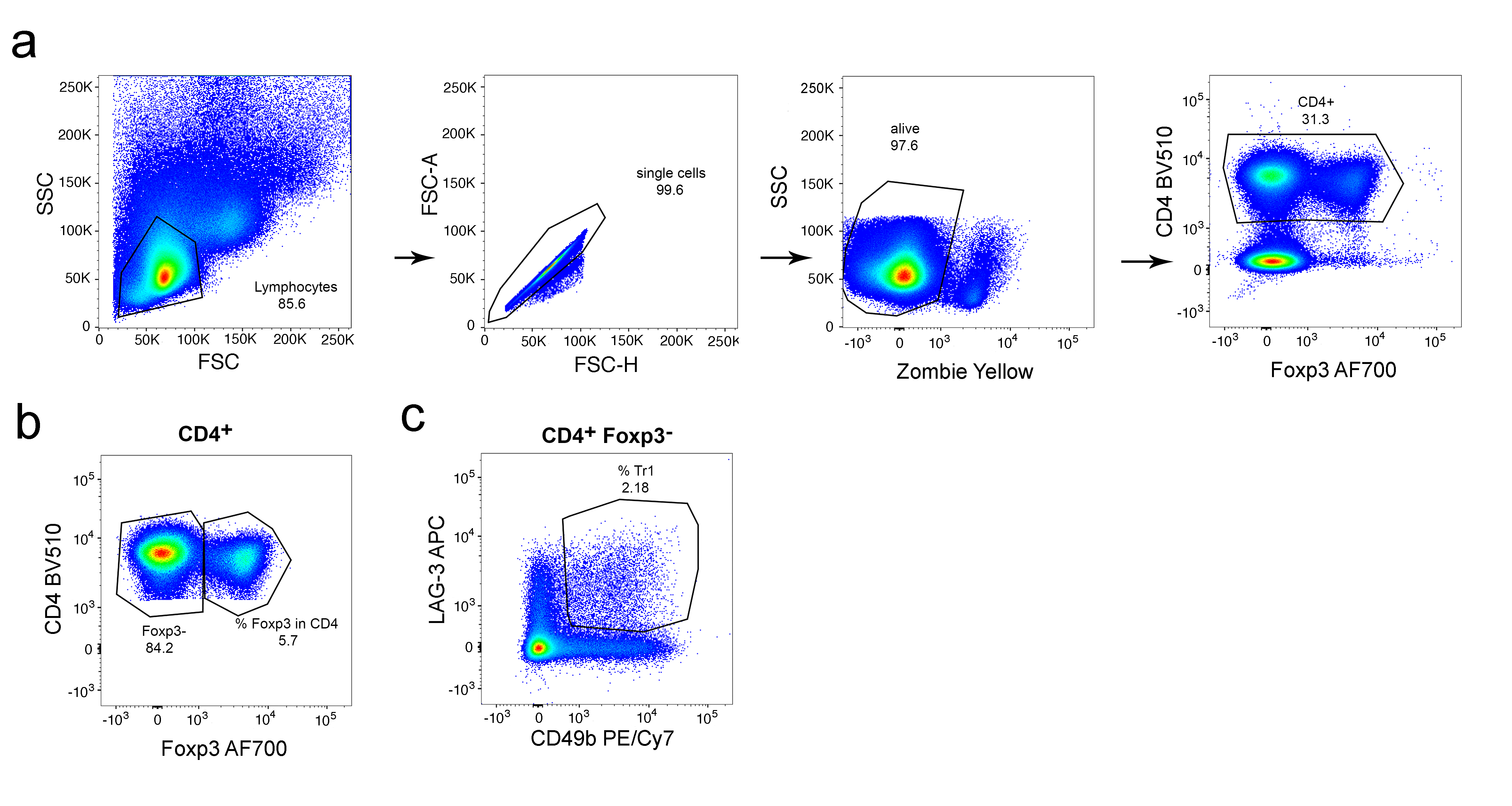 |
| --- |
| **Supplementary Figure 1:** C57BL/6 mice were infected with 2000 iL3. Six days later mesenteric lymph node cells were isolated and stained for CD4, Foxp3 and several checkpoint receptors. a) Representative dot blots showing the gating strategy (a) to differentiate CD4^+^ Foxp3^+^ Treg (b) and CD4^+^ Foxp3^-^ T cells. c) Representative dot blots showing the expression of CD49b and LAG-3 to identify Tr1 cells in the mesenteric lymph nodes from day 6 infected C57BL/6 mice. |

| 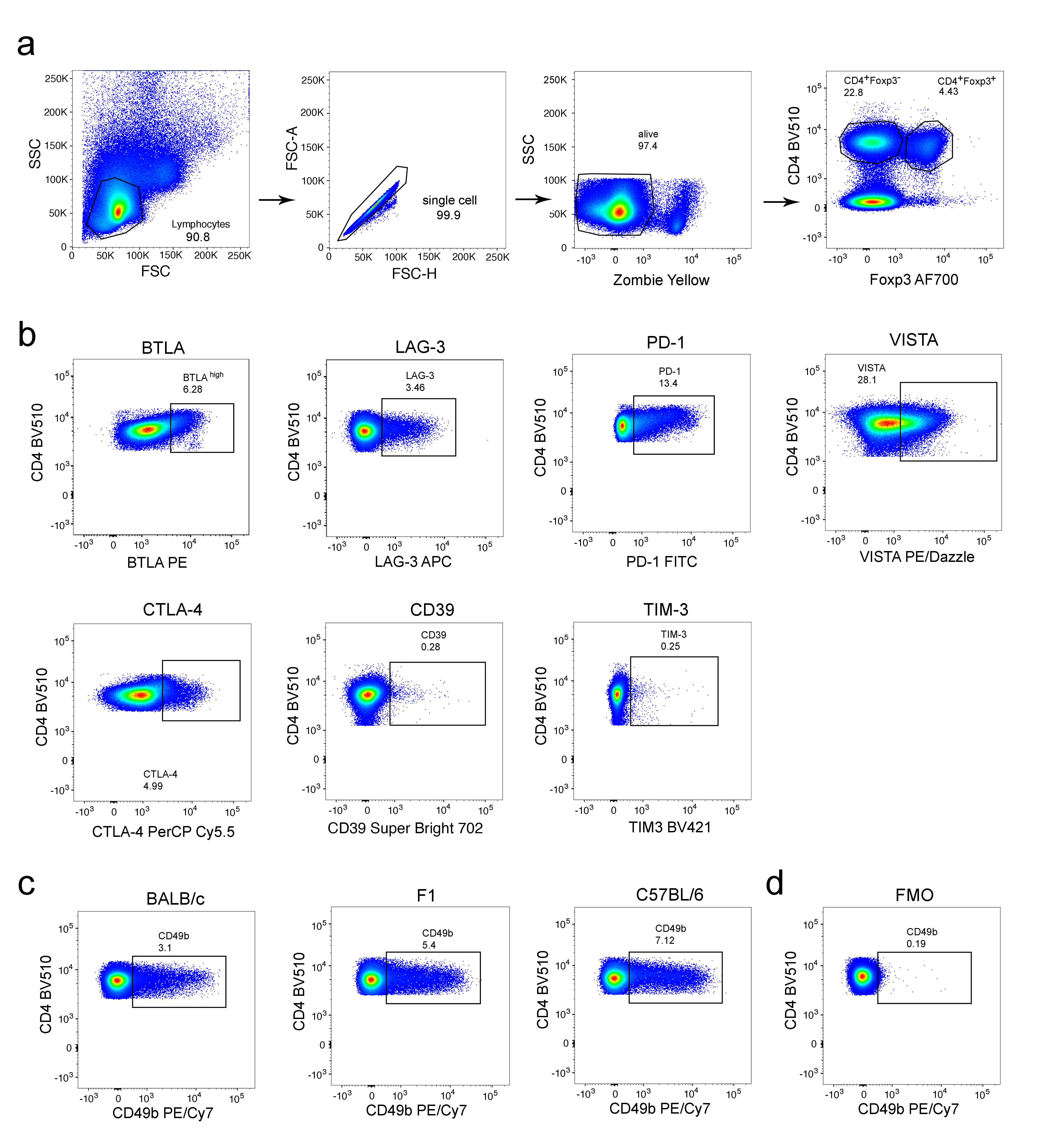 |
| --- |
| **Supplementary Figure 2:** C57BL/6 mice were infected with 2000 iL3. Six days later mesenteric lymph node cells were isolated and stained for CD4, Foxp3 and several checkpoint receptors. a) Representative dot blots showing the gating strategy to differentiate CD4^+^Foxp3^+^ Treg and CD4^+^Foxp3^-^ T cells. b) Representative dot blots showing the expression of BTLA, LAG-3, PD-1, VISTA, CTLA-4, CD39 and TIM-3 in the mesenteric lymph nodes from day 6 infected C57BL/6 mice. c) Representative dot blots showing the expression of CD49b in the mesenteric lymph nodes from day 6 infected BALB/c, F1 and C57BL/6 mice. d) Fluorescence minus one (FMO) control for the CD49b staining. |

| 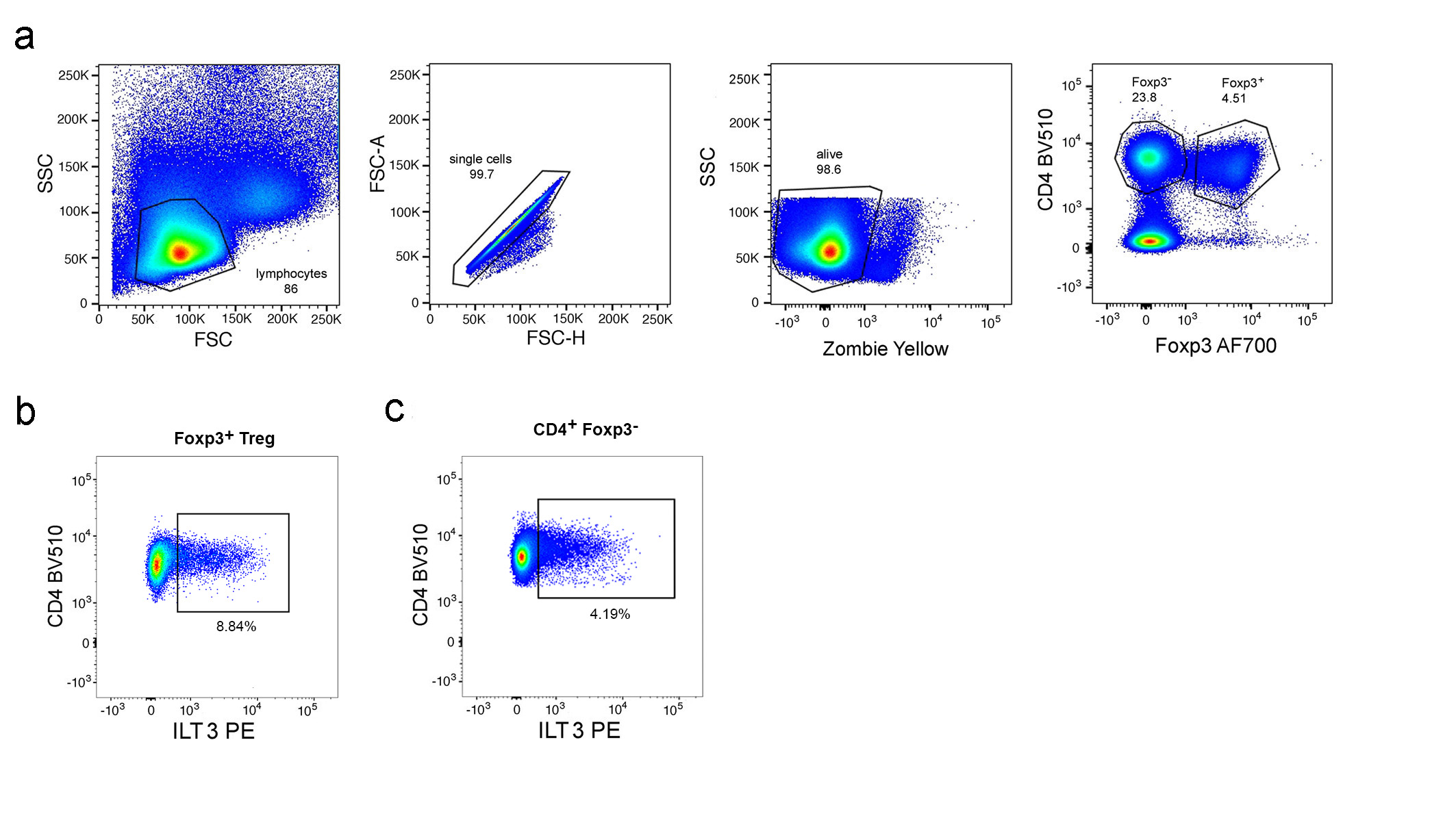 |
| --- |
| **Supplementary Figure 3: ILT3 gating** BALB/c, F1 and C57BL/6 mice were infected with 2000 iL3Six days later mes LN were isolated and stained for CD4 and ILT3 and intracellulary for Foxp3. a) Representative dot blots showing the gating strategy to distinguish Foxp3^+^ and Foxp3^-^ CD4^+^ T cells. b) Representative dot blot showing the ILT3 expression by (b) Foxp3^+^ Treg and Foxp3^-^ T cells from infected C57BL/6 mice. |
